# The *ansa*-bridged cyclo­penta­dienyl titanium complex [{η^5^-C_5_Me_4_CH_2_-C(NMe_2_)=N}TiCl_2_]

**DOI:** 10.1107/S1600536809015839

**Published:** 2009-05-07

**Authors:** Donglong Guo, Hong-Bo Tong, Meisu Zhou

**Affiliations:** a School of Life Science and Technology, Shanxi University, Taiyuan 030006, People’s Republic of China; b Institute of Applied Chemistry, Shanxi University, Taiyuan 030006, People’s Republic of China

## Abstract

The title complex, dichlorido[*N*,*N*-di­methyl-2-(η^5^-tetra­methyl­cyclo­penta­dien­yl)acetamidinido-κ*N*′]titanium(IV), [Ti(C_13_H_20_N_2_)Cl_2_], exhibits an unusual *ansa*-bridged conformation. The cyclo­penta­dienyl ring and the mean plane of the Ti—N=C—C—C fragment form a dihedral angle of 88.08 (11)°.

## Related literature

For related crystal structures, see: Hughes *et al.* (1993 ▶); Zhang *et al.* (2004 ▶). For general background, see: Chen & Marks (1997 ▶); Mahanthappa *et al.* (2004 ▶).
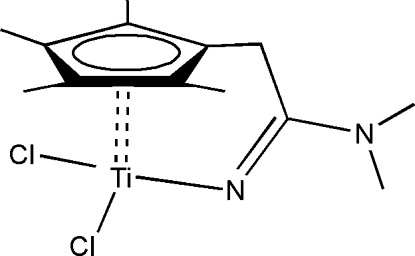


## Experimental

### 

#### Crystal data

[Ti(C_13_H_20_N_2_)Cl_2_]
*M* *_r_* = 323.11
Orthorhombic, 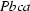

*a* = 12.600 (5) Å
*b* = 15.498 (6) Å
*c* = 15.574 (5) Å
*V* = 3041.1 (19) Å^3^
*Z* = 8
Mo *K*α radiation
μ = 0.90 mm^−1^
*T* = 213 K
0.30 × 0.20 × 0.20 mm

#### Data collection

Siemens SMART diffractometer
Absorption correction: multi-scan (*SADABS*; Sheldrick, 1997 ▶) *T*_min_ = 0.774, *T*_max_ = 0.841
11691 measured reflections
2677 independent reflections
2554 reflections with *I* > 2σ(*I*)
*R*_int_ = 0.037

#### Refinement

*R*[*F* ^2^ > 2σ(*F* ^2^)] = 0.056
*wR*(*F* ^2^) = 0.117
*S* = 1.27
2677 reflections
169 parameters
H-atom parameters constrained
Δρ_max_ = 0.38 e Å^−3^
Δρ_min_ = −0.30 e Å^−3^

### 

Data collection: *SMART* (Siemens, 1996 ▶); cell refinement: *SAINT* (Siemens, 1996 ▶); data reduction: *SAINT*; program(s) used to solve structure: *SHELXS97* (Sheldrick, 2008 ▶); program(s) used to refine structure: *SHELXL97* (Sheldrick, 2008 ▶); molecular graphics: *ORTEP-3* (Farrugia, 1997 ▶); software used to prepare material for publication: *SHELXL97*.

## Supplementary Material

Crystal structure: contains datablocks I, global. DOI: 10.1107/S1600536809015839/cv2554sup1.cif

Structure factors: contains datablocks I. DOI: 10.1107/S1600536809015839/cv2554Isup2.hkl

Additional supplementary materials:  crystallographic information; 3D view; checkCIF report

## Figures and Tables

**Fig. 1.**
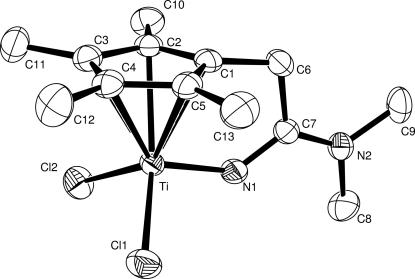
The molecular structure of (I), showing the atom-labeling scheme. Displacement ellipsoids are drawn at the 50% probability level. H atoms are omitted for clarity.

## supplementary crystallographic information

### Comment

The homogeneous coordination polymerization catalysts, especially group IV metallocene catalysts, have created new opportunities for the production of ethylene α-olefin copolymers and received extensive attention in recent years (Mahanthappa *et al.*, 2004). The constrained geometry catalysts with a pendant nitrogen or oxygen donor on the cyclopentadienyl ligand, such as Me_2_Si-(η^5^-Me_4_C_5_)(*t-*BuN)TiCl_2_ (Hughes *et al.*, 1993) and 2-tetramethylcyclopentadienyl-4-methylphenoxytitaniumdibenzyl (Zhang *et al.*, 2004) have been developed due to their structural features and good catalytic activities (Chen *et al.*, 1997). Here we present the synthesis and crystal structure of a new ansa-bridged cyclopentadienyl titanium complex (I)

In (I) (Fig. 1), the distance from the central metal atom Ti to the centroid of Cp* is 2.024 (2) Å. The bond lengths Ti—N1, Ti—Cl1 and Ti—Cl2 are 1.823 (3), 2.3104 (12) and 2.3036 (12) Å, respectively. The bond angle Cl1—Ti—Cl2 is 105.40 (5) °. Atoms C1, C6, C7, N1 and Ti are exactly co-planar with a highest deviation of 0.0191 Å. The two planes - Cp* and C1/C6/C7/N1/Ti are almost perpendicular making a  dihedral angle of 88.08 (11)°. The bond angles C1—C6—C7, C6—C7—N1 and C7—N1—Ti are 106.7 (3),116.7 (3) and 129.5 (2) °, respectively.

### Experimental

(CH_3_)_2_NCN (0.36 ml, 4.52 mmol) was added to a solution of PhN(Li)SiMe_3_(0.386 g, 2.26 mmol) in THF (30 cm^3^) at -78 °C. The resulting mixture was warmed to *ca*.25°C and stirred for overnight. CpTiCl_3_ (0.99 g, 4.52 mmol) was added at -78°C. The resulting mixture was warmed to *ca*.25°C and stirred for 24 h. The volatiles were removed in *vacuo*, and there residue was extracted with dichloromethane and filtered. The filtrate was concentrated to give red crystals of (I)(0.14 g, 13%).

Anal. calcd. for C_13_H_20_Cl_2_N_2_Ti(%): C, 48.33; H, 6.24; N, 8.67. Found: C, 48.25; H, 6.25; N, 8.73. A l l manipulations were performed under argonusing standard Schlenk and vacuum line techniques. THF was dried and distilled over Na underargon prior to use. Elemental analysis and NMR spectra are completely in agreement with the structure of (I). Spectroscopic analysis, ^1^HNMR (CDCl_3_): d 2.11~2.18 (d, 12 H, Cp—C*H*_3_), d 2.80, 3.10 (d, 6 H, N(C*H*_3_)_2_), d 4.09 (s, 2 H, C*H*_2_). ^13^CNMR (CDCl_3_): d 10.0, 10.8 (Cp-*C*H_3_), d 28.9 (*C*H_2_), d 33.3, 35.9 (N(*C*H_3_)_2_), d 118.5, 123.2, 127.4,128.6, 129.0 (Cp), 171.6 (CH_2_-*C*(NMe_2_)*-*N).

### Refinement

The H atoms were positioned geometrically and allowed to ride on their parent atoms, with C—H = 0.93-0.97 Å, and U_iso_ = 1.2-1.5 U_eq_(parent atom).

### Crystal data

**Table:**

[Ti(C_13_H_20_N_2_)Cl_2_]	*D*_x_ = 1.411 Mg m^−^^3^
*M**_r_* = 323.11	Mo *K*α radiation, λ = 0.71073 Å
Orthorhombic, *P**b**c**a*	Cell parameters from 4409 reflections
*a* = 12.600 (5) Å	θ = 2.5–27.0°
*b* = 15.498 (6) Å	µ = 0.90 mm^−^^1^
*c* = 15.574 (5) Å	*T* = 213 K
*V* = 3041.1 (19) Å^3^	Block, orange
*Z* = 8	0.30 × 0.20 × 0.20 mm
*F*(000) = 1344	

### Data collection

**Table:**

Siemens SMART diffractometer	2677 independent reflections
Radiation source: fine-focus sealed tube	2554 reflections with *I* > 2σ(*I*)
graphite	*R*_int_ = 0.037
ω scans	θ_max_ = 25.0°, θ_min_ = 2.5°
Absorption correction: multi-scan (*SADABS*; Sheldrick, 1997)	*h* = −14→14
*T*_min_ = 0.774, *T*_max_ = 0.841	*k* = −18→12
11691 measured reflections	*l* = −18→18

### Refinement

**Table:**

Refinement on *F*^2^	Primary atom site location: structure-invariant direct methods
Least-squares matrix: full	Secondary atom site location: difference Fourier map
*R*[*F*^2^ > 2σ(*F*^2^)] = 0.056	Hydrogen site location: inferred from neighbouring sites
*wR*(*F*^2^) = 0.117	H-atom parameters constrained
*S* = 1.27	*w* = 1/[σ^2^(*F*_o_^2^) + (0.036*P*)^2^ + 4.3005*P*] where *P* = (*F*_o_^2^ + 2*F*_c_^2^)/3
2677 reflections	(Δ/σ)_max_ = 0.001
169 parameters	Δρ_max_ = 0.38 e Å^−^^3^
0 restraints	Δρ_min_ = −0.30 e Å^−^^3^

### Special details

**Table:**

Geometry. All e.s.d.'s (except the e.s.d. in the dihedral angle between two l.s. planes) are estimated using the full covariance matrix. The cell e.s.d.'s are taken into account individually in the estimation of e.s.d.'s in distances, angles and torsion angles; correlations between e.s.d.'s in cell parameters are only used when they are defined by crystal symmetry. An approximate (isotropic) treatment of cell e.s.d.'s is used for estimating e.s.d.'s involving l.s. planes.
Refinement. Refinement of *F*^2^ against ALL reflections. The weighted *R*-factor *wR* and goodness of fit *S* are based on *F*^2^, conventional *R*-factors *R* are based on *F*, with *F* set to zero for negative *F*^2^. The threshold expression of *F*^2^ > σ(*F*^2^) is used only for calculating *R*-factors(gt) *etc*. and is not relevant to the choice of reflections for refinement. *R*-factors based on *F*^2^ are statistically about twice as large as those based on *F*, and *R*- factors based on ALL data will be even larger.

### Fractional atomic coordinates and isotropic or equivalent isotropic displacement parameters (Å^2^)

**Table:**

	*x*	*y*	*z*	*U*_iso_*/*U*_eq_	
Ti	0.21528 (5)	0.56734 (4)	0.10562 (4)	0.02449 (19)	
Cl1	0.10277 (7)	0.66810 (6)	0.04506 (6)	0.0396 (3)	
Cl2	0.11570 (8)	0.49802 (7)	0.20873 (6)	0.0416 (3)	
N1	0.3122 (2)	0.63161 (19)	0.16445 (18)	0.0302 (7)	
N2	0.4755 (2)	0.69295 (19)	0.19627 (19)	0.0326 (7)	
C1	0.3752 (3)	0.5272 (2)	0.0479 (2)	0.0289 (8)	
C2	0.3304 (3)	0.4522 (2)	0.0835 (2)	0.0285 (8)	
C3	0.2387 (3)	0.4307 (2)	0.0348 (2)	0.0314 (8)	
C4	0.2270 (3)	0.4933 (2)	−0.0298 (2)	0.0313 (8)	
C5	0.3117 (3)	0.5534 (2)	−0.0221 (2)	0.0299 (8)	
C6	0.4650 (3)	0.5788 (2)	0.0867 (2)	0.0347 (9)	
H6A	0.5014	0.6123	0.0423	0.042*	
H6B	0.5166	0.5403	0.1141	0.042*	
C7	0.4158 (3)	0.6384 (2)	0.1528 (2)	0.0282 (8)	
C8	0.4285 (3)	0.7477 (3)	0.2621 (3)	0.0458 (10)	
H8A	0.3529	0.7362	0.2658	0.069*	
H8B	0.4396	0.8078	0.2473	0.069*	
H8C	0.4615	0.7356	0.3171	0.069*	
C9	0.5892 (3)	0.7047 (3)	0.1806 (3)	0.0447 (10)	
H9A	0.6176	0.6536	0.1531	0.067*	
H9B	0.6253	0.7141	0.2348	0.067*	
H9C	0.5999	0.7544	0.1437	0.067*	
C10	0.3711 (3)	0.4014 (3)	0.1590 (2)	0.0421 (10)	
H10A	0.4040	0.3486	0.1387	0.063*	
H10B	0.3125	0.3872	0.1968	0.063*	
H10C	0.4229	0.4355	0.1900	0.063*	
C11	0.1708 (3)	0.3522 (2)	0.0475 (3)	0.0442 (10)	
H11A	0.0967	0.3678	0.0412	0.066*	
H11B	0.1826	0.3290	0.1046	0.066*	
H11C	0.1892	0.3090	0.0050	0.066*	
C12	0.1436 (3)	0.4946 (3)	−0.0985 (3)	0.0448 (10)	
H12A	0.1733	0.4725	−0.1516	0.067*	
H12B	0.1194	0.5534	−0.1073	0.067*	
H12C	0.0842	0.4588	−0.0812	0.067*	
C13	0.3304 (3)	0.6293 (3)	−0.0800 (2)	0.0438 (10)	
H13A	0.3836	0.6667	−0.0547	0.066*	
H13B	0.2647	0.6611	−0.0872	0.066*	
H13C	0.3550	0.6091	−0.1355	0.066*	

### Atomic displacement parameters (Å^2^)

**Table:**

	*U*^11^	*U*^22^	*U*^33^	*U*^12^	*U*^13^	*U*^23^
Ti	0.0217 (3)	0.0269 (3)	0.0249 (3)	0.0006 (3)	0.0008 (2)	−0.0016 (3)
Cl1	0.0328 (5)	0.0388 (5)	0.0471 (6)	0.0091 (4)	−0.0028 (4)	0.0043 (4)
Cl2	0.0404 (5)	0.0474 (6)	0.0370 (5)	−0.0053 (5)	0.0098 (4)	0.0072 (4)
N1	0.0284 (16)	0.0325 (16)	0.0296 (15)	0.0011 (13)	0.0017 (13)	−0.0100 (13)
N2	0.0275 (16)	0.0341 (17)	0.0362 (17)	−0.0027 (13)	−0.0056 (13)	−0.0058 (14)
C1	0.0254 (18)	0.0333 (19)	0.0281 (18)	0.0029 (15)	0.0048 (14)	−0.0097 (16)
C2	0.0273 (18)	0.0292 (18)	0.0292 (18)	0.0055 (15)	0.0010 (15)	−0.0057 (15)
C3	0.0316 (19)	0.0304 (19)	0.0322 (19)	0.0039 (16)	−0.0029 (16)	−0.0050 (16)
C4	0.034 (2)	0.0330 (19)	0.0270 (18)	0.0037 (16)	−0.0009 (15)	−0.0057 (16)
C5	0.0337 (19)	0.0284 (18)	0.0275 (18)	0.0008 (16)	0.0073 (15)	−0.0039 (15)
C6	0.0242 (18)	0.040 (2)	0.040 (2)	−0.0016 (16)	0.0036 (16)	−0.0100 (17)
C7	0.0259 (18)	0.0284 (18)	0.0302 (19)	0.0013 (15)	−0.0026 (15)	−0.0004 (15)
C8	0.049 (2)	0.041 (2)	0.047 (2)	−0.002 (2)	−0.010 (2)	−0.018 (2)
C9	0.035 (2)	0.045 (2)	0.054 (3)	−0.0144 (19)	−0.0090 (19)	0.000 (2)
C10	0.044 (2)	0.041 (2)	0.041 (2)	0.0107 (19)	−0.0116 (19)	0.0001 (19)
C11	0.045 (2)	0.034 (2)	0.054 (3)	−0.0069 (19)	−0.009 (2)	−0.0018 (19)
C12	0.052 (3)	0.049 (2)	0.034 (2)	−0.003 (2)	−0.0164 (19)	−0.0002 (19)
C13	0.053 (3)	0.042 (2)	0.036 (2)	−0.001 (2)	0.0120 (19)	0.0009 (18)

### Geometric parameters (Å, °)

**Table:**

Ti—N1	1.823 (3)	C6—C7	1.515 (5)
Ti—C1	2.292 (3)	C6—H6A	0.9800
Ti—Cl2	2.3036 (12)	C6—H6B	0.9800
Ti—Cl1	2.3104 (12)	C8—H8A	0.9700
Ti—C2	2.325 (3)	C8—H8B	0.9700
Ti—C5	2.341 (3)	C8—H8C	0.9700
Ti—C4	2.405 (3)	C9—H9A	0.9700
Ti—C3	2.406 (4)	C9—H9B	0.9700
N1—C7	1.322 (4)	C9—H9C	0.9700
N2—C7	1.319 (4)	C10—H10A	0.9700
N2—C8	1.457 (5)	C10—H10B	0.9700
N2—C9	1.464 (5)	C10—H10C	0.9700
C1—C2	1.406 (5)	C11—H11A	0.9700
C1—C5	1.412 (5)	C11—H11B	0.9700
C1—C6	1.512 (5)	C11—H11C	0.9700
C2—C3	1.422 (5)	C12—H12A	0.9700
C2—C10	1.504 (5)	C12—H12B	0.9700
C3—C4	1.405 (5)	C12—H12C	0.9700
C3—C11	1.500 (5)	C13—H13A	0.9700
C4—C5	1.422 (5)	C13—H13B	0.9700
C4—C12	1.500 (5)	C13—H13C	0.9700
C5—C13	1.500 (5)		
			
N1—Ti—C1	75.92 (13)	C5—C4—Ti	70.10 (19)
N1—Ti—Cl2	105.63 (10)	C12—C4—Ti	125.2 (3)
C1—Ti—Cl2	128.71 (10)	C1—C5—C4	107.5 (3)
N1—Ti—Cl1	104.29 (10)	C1—C5—C13	126.9 (3)
C1—Ti—Cl1	124.25 (10)	C4—C5—C13	125.5 (3)
Cl2—Ti—Cl1	105.40 (5)	C1—C5—Ti	70.40 (19)
N1—Ti—C2	94.34 (13)	C4—C5—Ti	75.1 (2)
C1—Ti—C2	35.44 (13)	C13—C5—Ti	121.3 (2)
Cl2—Ti—C2	94.88 (10)	C1—C6—C7	106.7 (3)
Cl1—Ti—C2	147.26 (9)	C1—C6—H6A	110.4
N1—Ti—C5	97.46 (13)	C7—C6—H6A	110.4
C1—Ti—C5	35.48 (12)	C1—C6—H6B	110.4
Cl2—Ti—C5	146.31 (9)	C7—C6—H6B	110.4
Cl1—Ti—C5	91.94 (10)	H6A—C6—H6B	108.6
C2—Ti—C5	58.66 (12)	N2—C7—N1	122.9 (3)
N1—Ti—C4	131.33 (13)	N2—C7—C6	120.4 (3)
C1—Ti—C4	58.19 (12)	N1—C7—C6	116.7 (3)
Cl2—Ti—C4	114.96 (10)	N2—C8—H8A	109.5
Cl1—Ti—C4	90.13 (9)	N2—C8—H8B	109.5
C2—Ti—C4	57.72 (12)	H8A—C8—H8B	109.5
C5—Ti—C4	34.83 (12)	N2—C8—H8C	109.5
N1—Ti—C3	128.99 (13)	H8A—C8—H8C	109.5
C1—Ti—C3	58.23 (12)	H8B—C8—H8C	109.5
Cl2—Ti—C3	88.61 (10)	N2—C9—H9A	109.5
Cl1—Ti—C3	118.89 (9)	N2—C9—H9B	109.5
C2—Ti—C3	34.93 (12)	H9A—C9—H9B	109.5
C5—Ti—C3	57.70 (12)	N2—C9—H9C	109.5
C4—Ti—C3	33.97 (12)	H9A—C9—H9C	109.5
C7—N1—Ti	129.5 (2)	H9B—C9—H9C	109.5
C7—N2—C8	120.2 (3)	C2—C10—H10A	109.5
C7—N2—C9	123.5 (3)	C2—C10—H10B	109.5
C8—N2—C9	116.3 (3)	H10A—C10—H10B	109.5
C2—C1—C5	108.4 (3)	C2—C10—H10C	109.5
C2—C1—C6	125.4 (3)	H10A—C10—H10C	109.5
C5—C1—C6	125.5 (3)	H10B—C10—H10C	109.5
C2—C1—Ti	73.55 (19)	C3—C11—H11A	109.5
C5—C1—Ti	74.12 (19)	C3—C11—H11B	109.5
C6—C1—Ti	110.9 (2)	H11A—C11—H11B	109.5
C1—C2—C3	108.0 (3)	C3—C11—H11C	109.5
C1—C2—C10	127.2 (3)	H11A—C11—H11C	109.5
C3—C2—C10	124.8 (3)	H11B—C11—H11C	109.5
C1—C2—Ti	71.01 (19)	C4—C12—H12A	109.5
C3—C2—Ti	75.7 (2)	C4—C12—H12B	109.5
C10—C2—Ti	119.9 (2)	H12A—C12—H12B	109.5
C4—C3—C2	107.8 (3)	C4—C12—H12C	109.5
C4—C3—C11	126.4 (3)	H12A—C12—H12C	109.5
C2—C3—C11	125.7 (3)	H12B—C12—H12C	109.5
C4—C3—Ti	73.0 (2)	C5—C13—H13A	109.5
C2—C3—Ti	69.41 (19)	C5—C13—H13B	109.5
C11—C3—Ti	125.7 (3)	H13A—C13—H13B	109.5
C3—C4—C5	108.3 (3)	C5—C13—H13C	109.5
C3—C4—C12	126.4 (3)	H13A—C13—H13C	109.5
C5—C4—C12	125.2 (3)	H13B—C13—H13C	109.5
C3—C4—Ti	73.1 (2)		
